# A Comprehensive Entomological, Serological and Molecular Study of 2013 Dengue Outbreak of Swat, Khyber Pakhtunkhwa, Pakistan

**DOI:** 10.1371/journal.pone.0147416

**Published:** 2016-02-05

**Authors:** Jehangir Khan, Inamullah Khan, Ibne Amin

**Affiliations:** 1 Zoology Department, Abdul Wali Khan University Mardan (AWKUM), Bunir Campus, Khyber Pakhtunkhwa (KPK), Pakistan; 2 Nuclear Institute of Food and Agriculture (NIFA), G.T Road, Tarnab Peshawar, Pakistan; Oswaldo Cruz Foundation, BRAZIL

## Abstract

**Background:**

*Aedes aegypti* and *Aedes albopictus* play a fundamental role in transmission of dengue virus to humans. A single infected *Aedes* mosquito is capable to act as a reservoir/amplifier host for dengue virus and may cause epidemics via horizontal and vertical modes of dengue virus (DENV) transmission. The present and future dengue development can be clarified by understanding the elements which help the dissemination of dengue transmission. The current study deals with molecular surveillance of dengue in addition to ecological and social context of 2013 dengue epidemics in Swat, Pakistan.

**Methods:**

Herein, we reported dengue vectors surveillance in domestic and peridomistic containers in public and private places in 7 dengue epidemic-prone sites in District Swat, Pakistan from July to November 2013. Using the Flaviviruses genus-specific reverse transcriptase (RT) semi nested-PCR assay, we screened blood samples (N = 500) of dengue positive patients, 150 adult mosquito pools and 25 larval pools.

**Results:**

The 34 adult and 7 larval mosquito pools were found positive. The adult positive pools comprised 30 pools of Ae. aegypti and 4 pools of Ae. albopictus, while among the 7 larval pools, 5 pools of Ae. aegypti and 2 pools of Ae. albopictus were positive. The detected putative genomes of dengue virus were of DENV-2 (35% in 14 mosquito pools & 39% in serum) and DENV-3 (65% in 27 mosquito pools & 61% in serum). The higher vector density and dengue transmission rate was recorded in July and August (due to favorable conditions for vector growth). About 37% of Ae. aegpti and 34% Ae. albopictus mosquitoes were collected from stagnant water in drums, followed by drinking water tanks (23% & 26%), tires (20% & 18%) and discarded containers (10% & 6%). Among the surveyed areas, Saidu was heavily affected (26%) by dengue followed by Kanju (20% and Landikas (12%). The maximum infection was observed in the age group of <15 (40%) followed by 15–45 (35%) and >45 (25%) years and was more in males (55.3%) as compare to females (44.7%). The increase in vector mosquito density and the subsequent viral transmission was determined by a complex interplay of ecological, biological and social factors.

**Conclusion:**

The suitable environmental conditions and discriminable role of *Aedes* through trans-ovarial transmission of DENV is indispensable in the recent geographic increase of dengue in Pakistan. Climate change affects the survival and dispersion of vectors as well as the transmission rates of dengue. Control of *Aedes* mosquitoes (vectors) and elimination of breeding sources must be emphasized and prioritized. Such actions may not only reduce the risk of dengue transmission during epidemics, but also minimize the chances of dengue viruses establishment in new (non endemic) areas of the region.

## Introduction

Internationally, dengue is regarded as the most important arboviral disease transmitted by mosquito. It is estimated that over 50% of the world’s population lives in areas where they are at risk of the disease, and approximately 50% live in dengue endemic areas [1–3]. Currently, there are 50 to 200 million dengue incidences worldwide with 500,000 cases of dengue hemorrhagic fever /dengue shock syndrome, and more than 20,000 deaths per annum [4].

In Pakistan, dengue is one of the emerging major public health concerns since 2005, leaving millions of lives at risk. Historically, dengue was first detected in 1994 in Karachi (southern part of Pakistan) and till October 2014, 48910 cases of dengue were recorded in Pakistan with 566 deaths. Several devastating outbreaks occurred during this period, but the first deadly outbreak was reported in Lahore (eastern part of Pakistan) in 2011, where 21,685 cases with 350 deaths were recorded [2,5–9]. Unprecedentedly, just one year after the major outbreak in Lahore, another massive outbreak (6,000 confirmed cases with 48 deaths) was recorded in the western part of Pakistan i.e. district Swat of Khyber Pakhtunkhwa (KPK).

Dengue is caused by dengue virus (DENV) with serotypesDENV-1, DENV-2, DENV-3 and DENV-4 and belongs to the family Flaviviridae and genus Flavivirus [10,11]. These serotypes can be transmitted to host through two vectors i.e. *Ae*. *aegypt* and *Ae*. *albopictus*. Adult female *Aedes* acquires the virus by biting an infected person during the viremic phase and transmit it to non infected persons via bites [12–14]. The best known mechanism of DENV transmission is horizontal (human-mosquito) transmission. However, trans-ovarial/vertical transmission [14], also provides a mechanism to understand how DENV persists in nature, i.e. in the absence of host or under unfavorable conditions for its vector’s activity [15]. Survival of *Aedes* mosquito eggs for relatively long periods of time (even more than a year) also allows the dengue virus to persist in the cold temperate and unfavorable environment for the adult vector [16,17].

The breeding sites (i.e. clean-water) of dengue vector are commonly found in the inner and outer domestic environments determined by human behavior [3,18,19]. In Pakistan, the major vector of dengue virus, *Ae*. *aegypti* has been introduced through tyres trade from India [1, 5, 20].

In spite of the fact that entomological surveillance provides critical background for better dengue disease management, in Pakistan systematic entomological surveillance for dengue vectors and their bionomics are the limitations [3]. As dengue has caused an alarming situation in the country, KPK in particular, we were intrigued to lay the foundation of this study to address the problem. The very essence of the present study is three dimensional, i.e. (i) to identify the potential breeding habitats and the factors facilitating the dispersion/breeding of vector mosquito; (ii) to determine the discriminable role of *Aedes* through the trans-ovarial transmission of dengue virus (DENV); (iii) to identify the circulating dengue serotypes found in 2013 dengue outbreak in district Swat through molecular and serological observations. The knowledge generated through this investigation will provide the technical basis for community-friendly preventive measures against dengue in Pakistan and elsewhere.

## Methods and Materials

### Study Area

Swat, a sub division of Malakand KPK, Pakistan, is a lush green valley situated in a mountainous range lying between 34^0^ 34" and 35^0^ 55" North latitudes and 72^0^ 08" and 72^0^ 50" East longitudes. It’s plain receives water from river Swat for irrigation which provides sufficient breeding grounds for vector mosquitoes. The climate of Swat is somewhat warm and humid with short and moderate summers; temperature seldom rises above 37°C. The annual rainfall averages around 33 inches with about 17 inches during June-September. The 2013 dengue epidemics were observed in lower Swat (Table 1) because of ideal climatic conditions for *Aedes* (dengue vector) growth. The total human population density of district Swat is 2,161,000 and a total of approximately 600 confirmed dengue cases with 48 deaths were reported from different Government as well as private hospitals in the district during 2013 dengue outbreak.

**Table 1:** Sampling areas visited for *Aedes* mosquito in district Swat.

S.no	Location	*Aedes aegypti*		*Aedes albopictus*	
		Adults (n = 2500)	Larvae (n = 450)	Adults (n = 500)	Larvae (n = 50)
1	Landikas (34°47′43.90″N,72°24′00.31″E)	300(12%)	59 (13%)	65 (13%)	6 (12%)
2	Gulkada (34°45′50.32″N, 72°21′54.54″E)	150 (6%)	27 (6%)	45 (9%)	5 (9%)
3	Rahim Abad (34°45′41.16″N,72°21′36.89″E)	350 (14%)	68 (15%)	90 (18%)	10 (19%)
4	Saidu (34°44′57.14″N, 72°21′23.03″E)	800 (32%)	148 (33%)	120(24%)	12 (25%)
5	Rang Mohala (34°46′26.01″N,72°21′51.82″E)	200 (8%)	31 (7%)	30 (6%)	3 (6%)
6	Kanju (34°49′45.32″N, 72°20′45.28″E)	500 (20%)	81 (18%)	100(20%)	9 (18%)
7	Amankot (34°45′51.79″N, 72°21′01.27″E)	200 (8%)	36 (8%)	50 (10%)	5 (11%)

### Study type and Sampling Strategies

**Fig 1:**
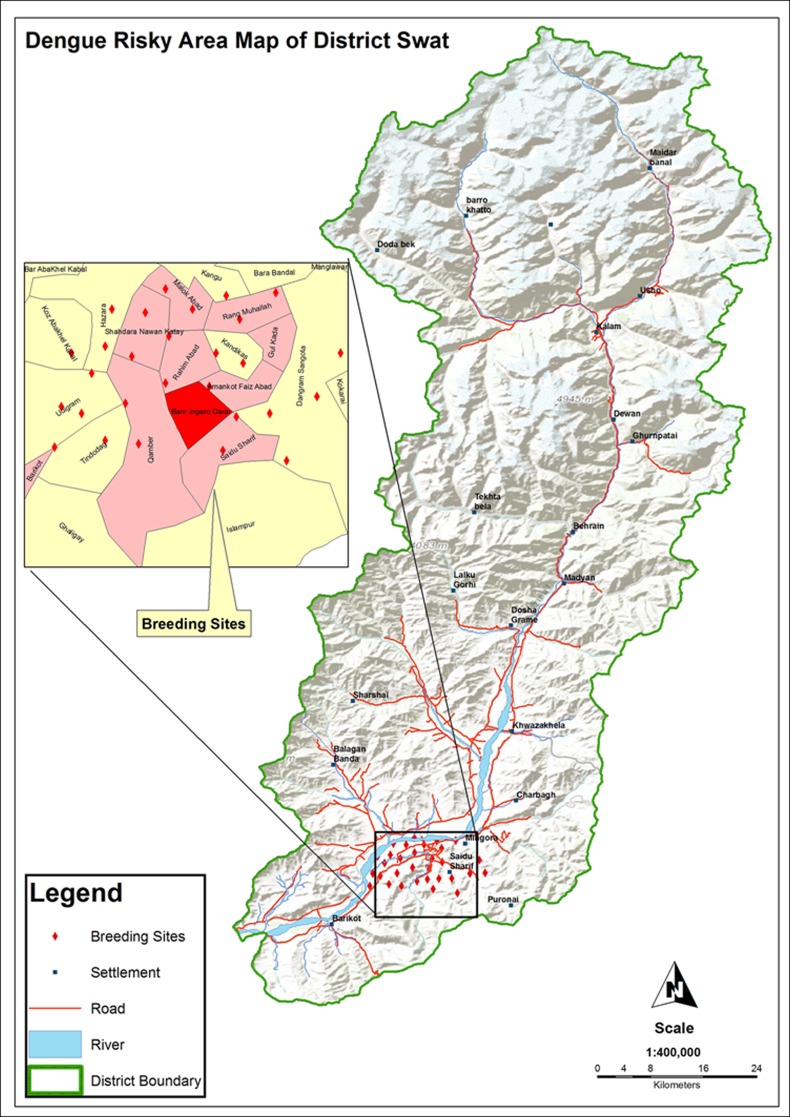
Map of district Swat showing the dengue prone sites and adjacent areas.

**Fig 2:**
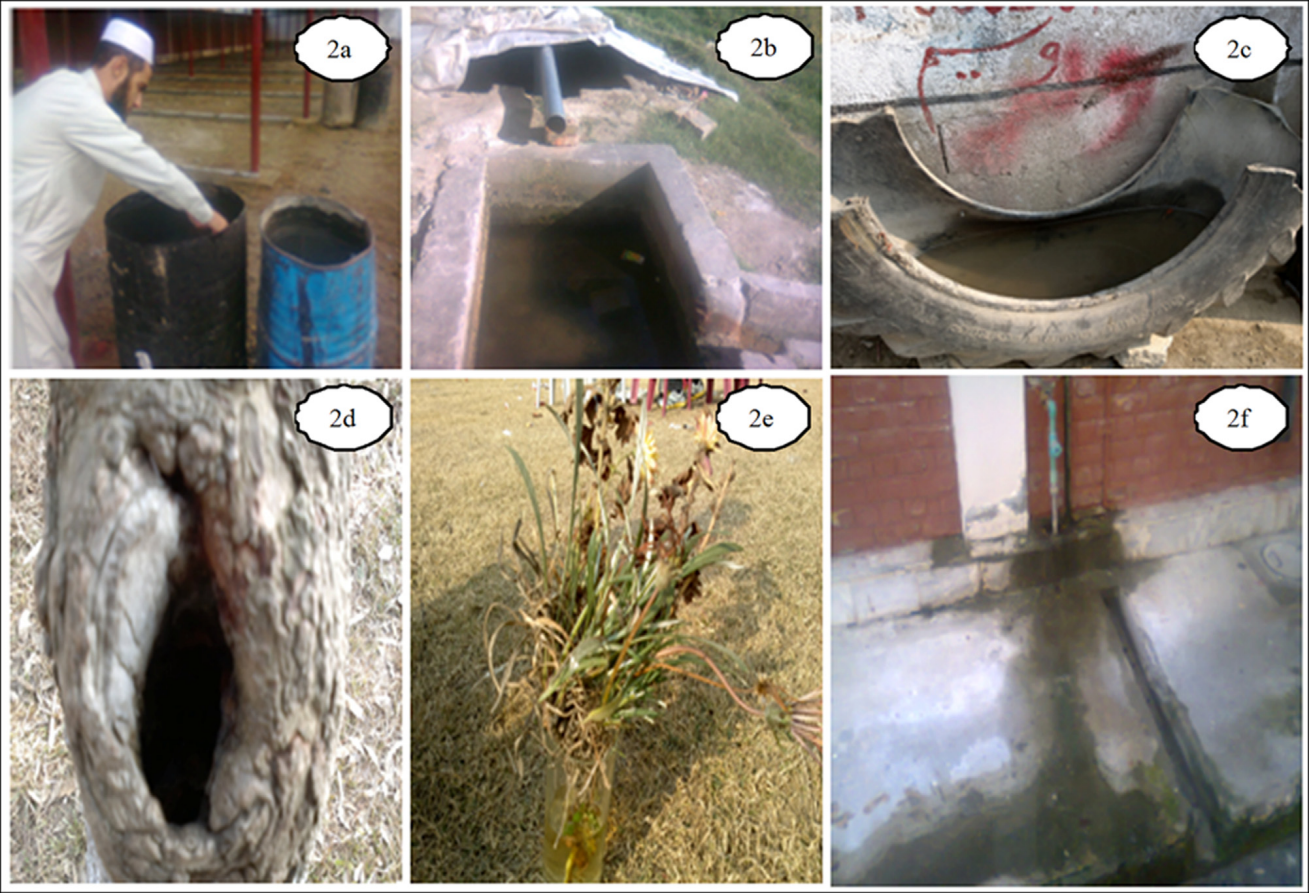
Different water containers and their physical shape during sampling. 2a: Mosquito larval collection from water drums containing water for construction purpose. 2b: Irrigation water tanks as a mosquito breeding places. 2c: Tire as a mosquito breeding sites. 2d: Plant hole as a habitat for mosquito. 2e: Guldasta (vessel) having fresh water acts as a best breeding site of *Aedes* mosquitoes. 2f: Leakage of water from water pipe due to poor sanitation has also provided the best opportunity for mosquito to breed.

The study is descriptive (entomological surveillance) and analytical (molecular detection of DENV in blood as well as in mosquitoes) in nature. All the seven dengue epidemic-prone sites (Fig 1, Table 1) of district Swat were visited for entomological survey during July-November 2013. The basic unit for sampling was water-holding containers (both manmade and natural) of the dengue patient’s home. Additionally, the potential outdoor breeding sites: tree holes, discarded small containers and used tyres (Fig 2) were also investigated. The collection was done in public as well as private places. Prior permission for sampling from privately own places was taken from their owners. As the field studies did not involve endangered/protected species or the protected/endangered areas, therefore, specific permission was not required for sampling. A total of 3000 adult *Aedes* mosquitoes (consisting of 2500 (83.4%) *Ae*. *aegypti* and 500 (16.6%) *Ae*. *albopictus*) and 500 larvae (consisting of 50 (10%) *Ae*. *albopictus* and 450 (90%) *Ae*. *agypt*: Tables 1 and 2) were captured from infested containers and backpack aspirator was used for adults. The specimens were preserved in 70% formalin and identified to species level by using the Leopoldo (2004) key [21].

**Table 2:** Collection of mosquitoes from different natural and man-made containers.

S.no	Habitats	*Aedes aegypti* (83.3%)		*Aedes albopictus* (16.6%)	
		Adults (n=2500)	Larvae (n=450)	Adults (n=500)	Larvae (n=50)
1.	Discarded containers	250 (10%)	36 (8%)	30 (6%)	3 (5%)
2.	Water Drums	925 (37%)	153 (34%)	170 (34%)	18 (36%)
3.	Plant vessels	125 (5%)	13 (3%)	30 (6%)	3 (5%)
4.	Tires	500 (20%)	135 (30%)	90 (18%)	7 (17%)
5.	Tree holes	125 (5%)	13 (3%)	50 (10%)	5 (9%)
6.	Water Tank	575 (23%)	100 (22%)	130 (26%)	14 (28%)

### Pools Formation

The entire 3000 adult mosquitoes were grinded in 150 pools consisting 125 (83%) pools of *Ae*. *aegypti* and 25 (17%) pools of *Ae*. *albopictus* (Table 3). The wild collected larvae were also grinded in 25 pools comprising 18 (72%) pools of *Ae*. *aegypti* and 7 (28%) pools of *Ae*. *albopictus* (Table 4).

**Table 3:** Distribution of dengue virus in adult pools of *Ae*. *aegypti* and *Ae*. *albopictus* from various sites in district Swat, Khyber Pakhtunkhwa, Pakistan.

Species & sites	No. of pools	Individuals/P*	Total individuals	PCR Positive Pools	MIR
***Aedes aegypti***	**125**	**20**	**2500**	**30 (24%)**	
Saidu	20		400	07	**17.5**
Amankot	15		300	03	**10**
Rang Mohalla	15		300	02	**6.6**
Landikas	20		400	04	**10**
Gulkada	20		400	03	**7.5**
Rahim Abad	20		400	06	**15**
Kanju	25		300	05	**6.6**
***Aedes albopictus***	**25**	**20**	**500**	**4 (16%)**	
Saidu	3		60	1	**16.6**
Amankot	1		20	--	--
Rang Mohala	4		80	--	--
Landikas	8		160	1	**6.25**
Gulkada	2		40	--	--
Rahim Abad	4		80	1	**12.5**
Kanju	3		60	1	**16.6**
**Total**	**150**		**3000**	**34 (22.6%)**	

*P means pool

**Table 4:** Distribution of dengue virus in larval pools of *Ae*. *aegypti* and *Ae*. *albopictus* from various sites in district Swat, Khyber Pakhtunkhwa, Pakistan.

Species & sites	No.of pools	Individuals/P*	Total individuals	PCR Positive Pools	MIR
***Aedes aegypti***	22	20	450	**5 (22.7%)**	
Amankot	04		80	--	--
Saidu	02		40	2	**50**
Rang Mohalla	02		80	--	**--**
Gulkada	04		80	1	**12.5**
Rahim Abad	03		60	1	**16.6**
Kanju	02		40	1	**25**
Landikas	01		40	--	**--**
***Aedes albopictus***	**07**			**2 (28.5%)**	
Rahim Abad	01		07	--	--
Gulkada	--		--	--	--
Rang Mohala	--		--	--	--
Landikas	01		07	--	--
Kanju	02		14	1	71
Saidu	02		14	1	71
Amankot	01		07	--	--
**Total**	**25**		**500**	7	**(28%)**

*P means pool

### Ethics Statement

The study and associated protocols were designed based on national ethical legislative rules and approved by Local Ethic Committees of AWKUM, Bunir Campus. All samples were collected after written consent of the relatives of individuals (blood donors) according to the updated version of the declaration of Helsinki [22]. The participant in the figure is the author himself and has given permission to publish his image.

### Blood sampling

The Saidu Group of teaching hospital, Shifa Medical Center, and Saidu Sharif Teaching hospitals of the district were visited on daily basis. Prior permission for obtaining patient history was taken from the Medical Superintendents (MSs) of the Dengue Ward in the hospitals. Four ml blood from each admitted dengue patients (IgG and IgM positive) was collected in EDTA tubes, the serum was isolated from the blood (n = 500) and preserved at -80°C. A questionnaire comprising multiple questions regarding the history, information about the disease and the patients (S1 File) was filled. The blood sampling was carried out within the first three days of illness. Similarly, the surveillance of *Aedes* mosquitoes in homes (n = 832) was carried out immediately after confirmation of dengue in patients admitted in hospitals (Table 5). Mostly the dengue infection was observed in the age group of <15 years followed by 15–45 and >45 years (Table 5).

**Table 5:** Age, sex and area wise distribution and the incidences of Dengue.

**Month wise dengue incidences**								
**Month**	**July**	**August**	**September**		**October**		**November**	
**Incidences**	1560 (26%)	1680 (28%)	1260 (21%)		900 (15%)		600 (10%)	
**Month wise increase/decrease of vector density**								
***Ae*. *aegypti***	21%	24%	22%		18%		15%	
***Ae*. *albopictus***	19%	24%	20%		19%		18%	
**Age/Sex wise distribution of Dengue Patients**								
<15 years		15–45 years			>45 years		Over all prevalence in male and female (%)	
40% (N = 2400)		35% (N = 2100)			25% (N = 1500)		Males: 3320 (55.3)	
1300 M	1100 F	1150 M	950 F		870 M	630 F	Females: 2680(44.7)	
**Surveyed houses and areas wise distribution of dengue incidences**								
Patients distribution	Saidu	Kanju		Rahim abad	Landikas	Rang mohalla	Amankot	Gulkada
	26% (N = 1560)	20% (N = 1200)		15% (N = 900)	12% (N = 720)	10% (N = 600)	9% (N = 540)	8% (N = 480)
Positive houses /Inspected Houses for *Aedes*	80/158	65/146		59/139	60/134	50/101	45/79	45/75

#### RNA extraction

A nested RT-PCR developed by [23] with minor modifications was used to analyze the mosquitoes and blood samples. We tried best to exclude the possible laboratory contamination, and provided additional data for future studies on the degree of variation in the genomic segment used. Samples (grinded mosquitoes/blood serum) (150 μl) were taken, and RNA was extracted with Favorgine RNA extraction kit (CAT# FAVNKOO1-2) according to the instructions of manufacturer. RNA (5 μl) was reverse transcribed, the cDNA (the C-prM junction of the dengue virus genome of 511 bp) was amplified with primers D_1_ (Upstream/Forward) (59-TCAATATGCTGAAACGCGCGAGAAACCG-39/InvitrogenH) and D_2_ (Downstream/Reverse) (59-TTGCACCAACAGTCAATGTCTTCAGGTTC-39/InvitrogenH) [23] using MMLV-reverse transcriptase (Fermentas, USA) in a single reaction vessel with 50 μl final volume. The thermocycler was programmed to incubate for 45 minutes at 42°C and then 35 cycles at 94°C for 30 seconds, 55°C for 1 minute, and 72°C for 2 minutes and 72°C for 5 minutes. Similarly, The second step of the nested-PCR was carried out with D1 and type-specific (TS) reverse primers (TS1:59CGTCTCAGTGATCCGGGGG3’; TS2:59CGCCACAAGGGCCATGAACAG3’;TS3:59TAACATCATCATGAGACAGAGC3’; TS4: 59 CTCTGTTGTCTTAAACAAGAGA), which amplify regions of 482, 119, 290 and 392 bp of DENV-1, DENV-2, DENV-3 and DENV-4, respectively [23]. The detection of amplified fragments was performed by gel electrophoresis (1.5% agarose gel stained with 1% ethidiumbromide).

#### Minimum Infection Rate (MIR)

MIR was calculated according to the key [24].

$$\mathrm{MIR}=\frac{\mathrm{number of positive pools by species}}{\mathrm{total number of that species tested}}x\;1000$$

## Results

### Entomological surveillance

**Fig 3:**
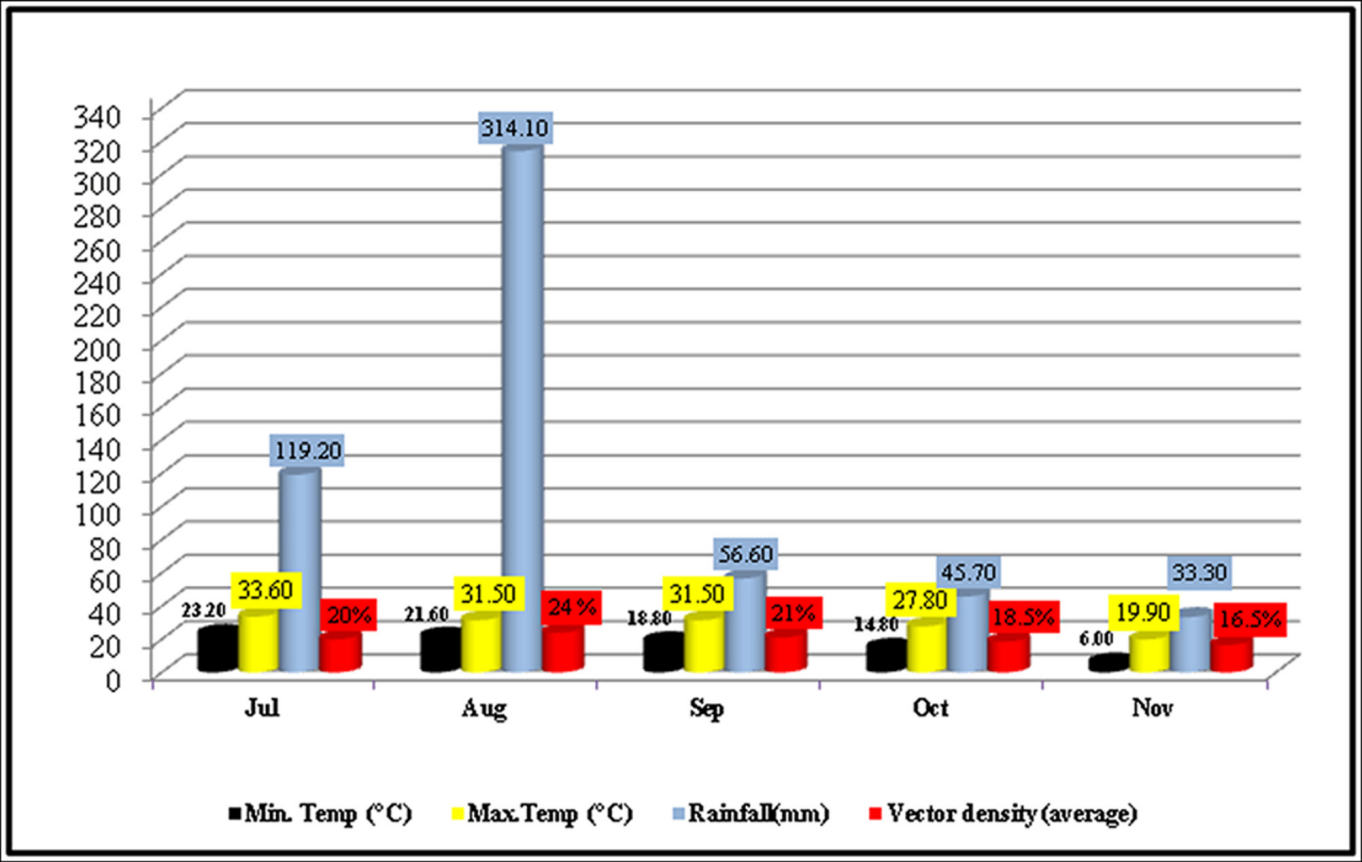
Relationship of rainfall and temperature on vector mosquito density.

Out of 832 inspected houses, 404 were houses were positive (Table 5), the *Ae*. *aegypti* and *Ae*. *albopictus* were collected from indoor and outdoor natural and manmade (domestic) water holding containers (Table 2; Fig 2). The wide spread distribution of this mosquito suggests that it has established in the district and can bring more severe epidemics in future again upon favorable conditions for its dispersion/breeding. The relative abundance of *Ae*. *albopictus* was found low (18.6%) as compared to *Ae*. *aegypti* (81.3%) (Tables 1 and 2). Our findings suggest that *Ae*. *albopictus* may not actively participated in causing the dengue epidemics in district Swat (2013). This may be due to its non-domestic behavior. The positivity of different water-holding containers (Fig 2) for vector mosquito has been shown in Table 2. Furthermore, the highest collection of mosquito was done in the month of August and lowest in November as shown (Fig 3 and Table 5).

### Ecological and Social factors affecting vector density/dispersion and dengue transmission

**Fig 4:**
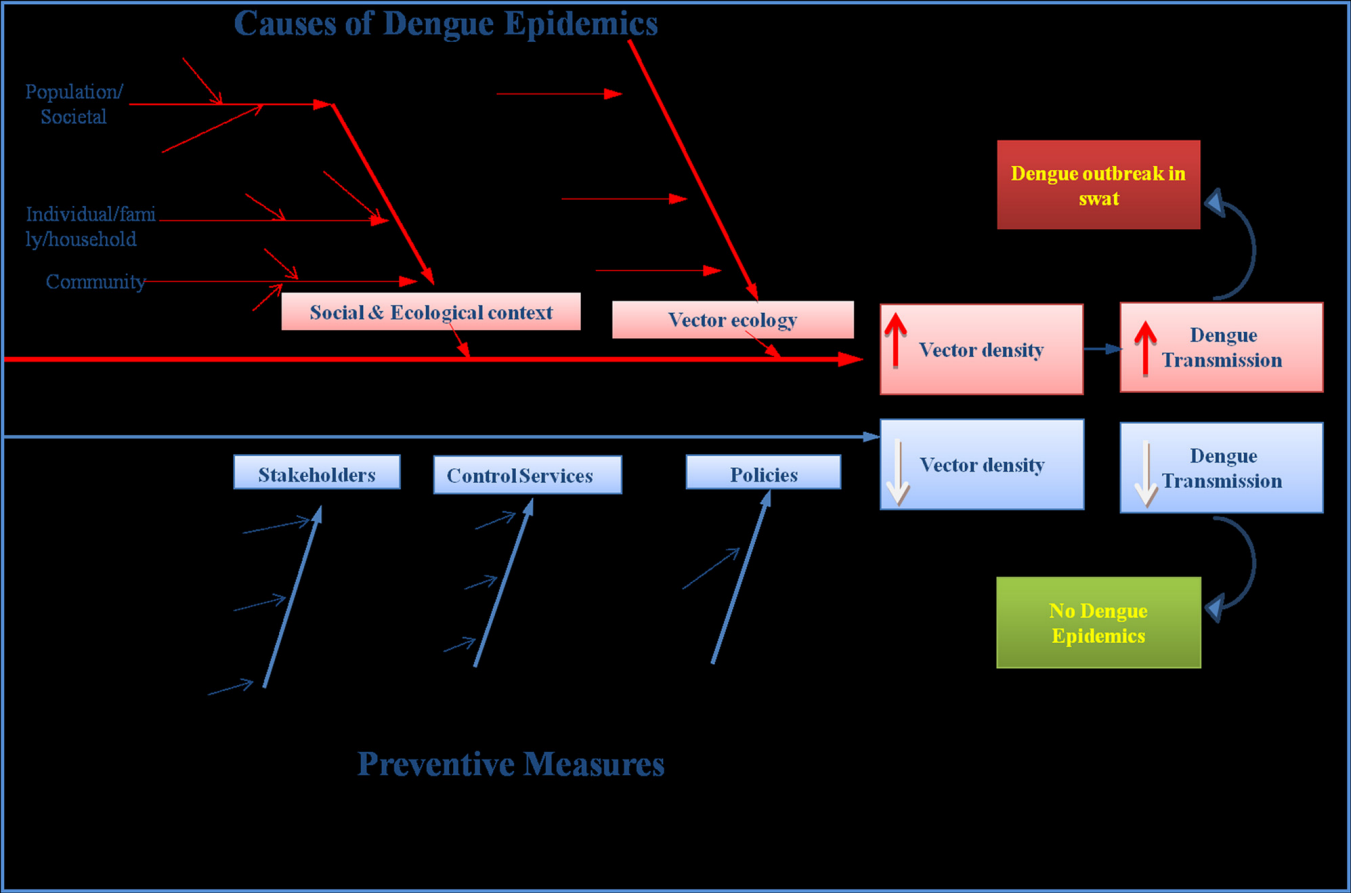
Eco-bio-social factors involved in Dengue epidemics in Swat: A Conceptual framework.

Several complex factors associated with dengue transmission were observed in the most urbanized cities (Kanju, Rahimabad and Saidu) of district Swat (Fig 1). The studied areas are occupied by maximum human populations as compared to rest of the surveyed areas. Unplanned urbanization and human population growth has resulted in inadequate water sewerage, waste management systems, substandard housing and poor sanitation might have led to the reproduction/dispersion of vector mosquitoes in Swat, which subsequently has increased the risk of dengue transmission to its inhabitants as explained explicitly (Fig 4). Similarly, the tires were also observed as the best breeding sites (Table 2, Fig 2) for *Aedes*. Moreover, the movement of dengue patients and the extensive trade of old tires contaminated with the DENV infected eggs of *Aedes* from Lahore (Punjab), Peshawar (KPK) and Karachi (Sindh) where recently the dengue epidemics were recorded, may have been a source of share of the dengue virus to district Swat. The interplay of human’s travel and the transmission of dengue has been known from the statements of some of the dengue patients who were bitten by dengue vector mosquito in Punjab but develop the symptoms when reached at their home in Swat. In this fashion a single person infected with dengue may become a reservoir of DENV for other uninfected mosquitoes and hence become the cause of a volley of dengue outbreak in an area.

**Fig 5:**
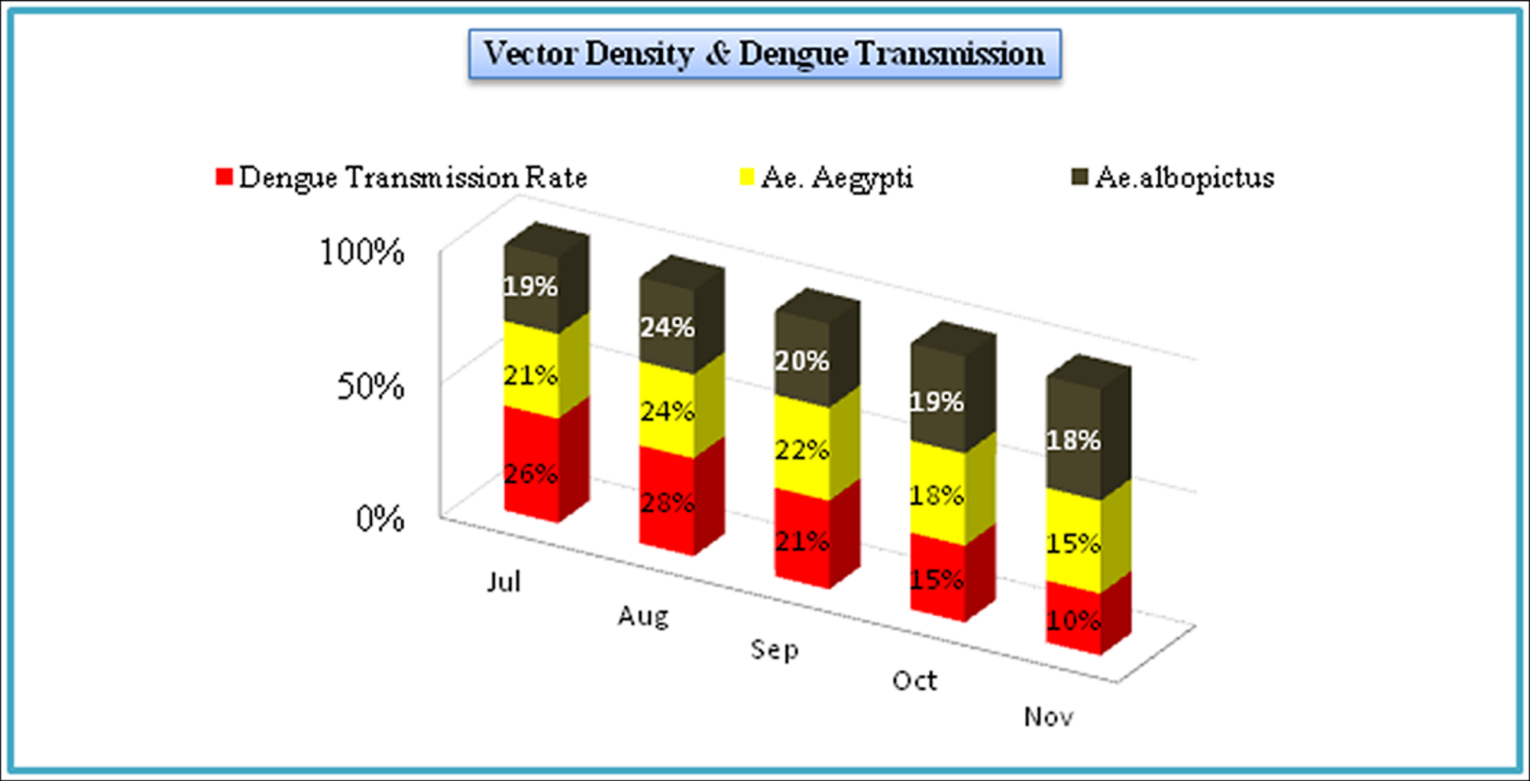
Dengue incidence and transmission rate in relation to vector density.

One of the important aspects of our findings is that we observed the impact of two factors, especially temperature and rain fall (humidity), on the vector dispersion/density. The higher (29–30 C°) temperature and the maximum humidity due to heavy rainfall in months of July-September (2013) overall favored the increase in vector (mosquito) population size as compared to late summer (October-November) (Fig 3 and Table 5). This extended vector density was one of the causes for frequent human-mosquito contacts, which subsequently caused a rising trend of dengue patients’ hospitalization in July (26%) followed by August (28%) and September (21%) (Figs 3 & 5, Table 5). The maximum vector density and the vectoral capacity of *Aedes* expanded the domain of this natural calamity and we observed, interestingly, two cases of dengue infection, one was the nursing faculty member and another was a student form Saidu Medical college living in the hostel, both were bitten by an infected mosquito and thus suffered from dengue. The travel trade, movement of dengue patients and the ecological/environmental factors have played a significant role in the dispersal of dengue and its vectors.

### Prevalence and distribution of DENV serotypes in mosquitoes and blood

Among 150 pools of adult mosquitoes, 30 pools of *Ae*. *aegypti* and 4 pools of *Ae*. *albopictus* were positive (Table 3). Out of 25 larval pools, the 7 pools were found positive comprised 5 pools of *Ae*. *aegypti* and 2 pools of *Ae*. *albopictus* (Table 4). Among adult mosquitoes, the DENV-2 was identified in 11 (32%) pools and DENV-3 in 22 (65%) pools, while a single (3%) pool showed the concurrent infection of DENV-2 & 3 (Tables 3 and 4). Similarly, among 7 larval positive pools, 2 (29%) pools showed the presence of DENV-2 and 5 (71%) pools showed the presence of DENV-3 (Tables 3, 4 & 6). The positive controls for DENV serotypes were available from 2012 dengue epidemics in Peshawar, KPK, when some of the DENV positive samples were preserved at -80°C for future studies. Additionally, the 350 blood samples were found positive for DENV after qualitative PCR. The positive samples (blood as well as mosquito) were further processed for genotyping using the type specific (TS1-TS4) primers. The overall results showed the presence of DENV-3 in 27 (65%) mosquito pools and 213 (61%) blood samples, the DENV-2 was identified in 11 (32%) mosquito pools & 130 (37%) blood samples. Our results have shown that DENV-3 is a major circulating serotype in Swat. Mix infections of DENV-2 & 3 were observed only in 7 (2%) blood samples and 1 (3%) pool of mosquito (Table 6). One of the interesting findings of the present research is the detection of similar serotypes of dengue in the blood serum as well as in mosquitoes collected from the patient’s home (Table 5). Similarly, the houses supporting more breeding places for mosquitoes were loaded with maximum *Aedes* population and subsequently with more dengue patients. The reason may be the frequent contact of mosquitoes with dwellers. Increased MIR values in mosquito were significantly associated with the increase dengue infections in humans.

**Table 6:** Comparison of serotypes distribution in mosquito (Adults & Larvae) and blood samples.

S.no	DENV	Blood	Adults mosquito pool	Larvae pool
1	Serotype 2	37%	32%	29%
2	Serotype 3	61%	65%	71%
3	Concurrent Infection With DENV-2 & 3	2%	3%	

## Discussion

Current evidence indicates that dengue is expanding its geographical range in Pakistan as well as in the rest of the world, causing increasing numbers of outbreaks associated with human morbidity and mortality. The present study is the first to explain the entomological, molecular, ecological and social context of dengue outbreak in district Swat with more than 6000 confirmed cases and 48 deaths.

The *Ae*. *aegypti*and *Ae*. *albopictus* are important vectors of DENV in South-East Asia. The former species is highly anthropophilic in nature and believed to rest inside houses [3, 25–31], whereas the latter prefers outdoor [14, 16, 28, 29]. The results of the present study confirmed the indoor preference of *Ae*. *aegypti* and their associations with the water storage containers in the houses, while the *Ae*. *albopictus* (larvae and adults) was mostly found in outdoor water drums and tires respectively (Fig 2A and 2C). Moreover, our results in agreement with other reported studies on association of mosquito’s with water tanks and drums (Fig 2A and 2B) [3, 30, 31] as the most attractive breeding sites for both species of the dengue vectors. The relative abundance of *Ae*. *albopictus* as noted in our study is only 16.6% of the total collection (Tables 2). This suggests for the frequent contact of *Ae*. *albopictus* with various wild vertebrates for a blood meal as other studies also [32–35] reported the similar trend.

In accordance with some previous findings [36–39], fascinatingly, our studies indicated a distinct positive association of *Ae*. *aegypti* particularly, and *Ae*. *albopictus* generally, with tires (Fig 2C). Additionally, a large proportion of water containers infested with larvae and/or pupae of *Aedes* were plant vessels (Fig 2E), small pots and cans, whereas the adults preferred large water tanks or barrels (Fig 2B), our results are congruent with other research studies [19, 40–42]. The presence of *Aedes* mosquito in tyres suggests a mean of transportation/dispersion of dengue vectors in and outside the country, and thus might be one of the causes of dengue outbreak in Swat. Some studies [43, 44] have reported *Ae*. *albopictus* as the efficient vector of dengue, while other [19] have mentioned the *Ae*. *aegypti*as a main culprit of dengue outbreaks. In our research, we found both *Ae*. *aegypti* (83.4%) and *Ae*. *albopictus* (16.6%) responsible for dengue outbreak of Swat and are, therefore, in close agreement with another national report [3]. Our results for the first time have confirmed that both the species of *Aedes* are involved in the transmission of dengue in Pakistan.

The hospitalized dengue patients and the mosquitoes having highest MIR (Tables 3 and 4) in Swat were belonging to the areas (Kanju, Saidu and Rahim Abad) (Fig 1, Table 5), where high load of *Aedes* was recorded. In these areas due to the irregular supply of electricity and drinking water, the local people are used to store water in uncovered drums and tanks (Fig 2A and 2B) and thus reflect a perfect site (Fig 2F) for the *Aedes* breeding. These conditions favored the increase of population size of this mosquito and subsequently led to its dispersion and frequent contacts with the local people and maximum transmission of dengue occurrence. This suggests that density of *Aedes* (infected with DENV) may act as an indicator of dengue transmission in a region. These results are congruent with international [43, 45–47] and national reports [3].

Analysis of dengue patient’s hospitalization and meteorological data (Table 5, Figs 3 & 5) revealed an imperative role of temperature in the rise of dengue incidence. The highest number of dengue patients during July to September (Figs 3 & 5) may be due to repeated feeding of *Aedes* mosquitoes on humans. Different studies [13, 48–50] in the past have shown that higher temperature (>25°C) produces large number of mosquitoes with frequent blood feeding nature. Also, it is documented that 1°C increase in temperature (above average) may increase risk of dengue transmission by 1.95 times [43, 51, 52]. Rainfall (humidity) is another ecological factor which makes an ideal condition for mosquito to breed and as a result its population density increases (Fig 3). Additionally, humans often stay indoors when it rains which increases the contact of *Ae*. *agypti* (specifically) to humans. Thus indoor stay of *Ae*. *aegypti* as well as humans due to high rainfall during monsoon period provides best opportunity for the DENV to be communicated/transmitted. This might be the reason in the present study that the increased hospitalization of dengue patients was recorded during the months (July, August and September) having maximum vector density (Figs 3 & 5, Table 5). Similar observations have been documented in previous studies [47, 53]. A number of studies have also demonstrated that the egg viability [54] and population size of the vector [55] increases in humid conditions. In Pakistan a series of flood after 2010, therefore, favored conditions for the dispersal of *Aedes* [56, 57] and subsequently Pakistan has suffered devastating outbreaks of dengue after 2010. This complex interplay of ecological (temperature and rain/humidity etc.), biological (DENV loaded mosquito/human displacement/travelling) and social factors (water storage, urbanization, waste disposal, cross-border travel & trade) (Fig 4) are some of the subsequent causes of vector dispersion and sudden outbreak of dengue in Swat. Similar observations have been documented by six other countries of Asia [13, 40]. Surveillance (minor observation) for dengue vectors in our study has revealed that various public places (colleges, schools, university, hospitals and grave yard) are also the major breeding sites for *Aede*s in Swat. An international report [40] has also confirmed this.

The current study has observed maximum dengue infection in human males (55.3%) as compared to females (44.7%), similarly, the individuals having age <15 years had highest (40%) infection followed by 15–45 (35%) and >45 (25%) years individuals (Table 5). Our results are in accordance with the published research reports [2, 4, 9]. The highest dengue infections in children may be due to their exposed body parts to *Aedes* for quick and easy blood meals. Moreover, the low dengue infection in females may be due to the minimum exposed body parts as compared to males, in KPK and especially in Pakhtun culture where women wear long arms shirts and scarp due to which they are fully covered from mosquito access. This study suggests the need of individual/self based protection from mosquito access specifically during the hot and rainy season.

The role of *Aedes* mosquito in transmission of DENV can be estimated through the minimum infection rates (MIRs), which may serve as a tool for predicting epidemics [15]. The *Aedes* mosquito eggs are capable to survive under adverse conditions for a long time and may become a source of dengue transmission around the globe [58]. Multiple studies [15, 59–68] on transmission of DENV via eggs have been documented. Our study found 41 pools out of 175 pools positive (Tables 3 and 4) for DENV recording the MIRs within the range reported previously [15, 69–74]. The DENV-2 & DENV-3 detected in Swat may have been introduced via transportation, extensive trade of tires etc, tourism among Lahore, Swat and Karachi, and internal migration of IDPs (internally displaced people due to terrorist attacks), because these two serotypes were initially detected in Karachi (DENV-2 in 1994 and DENV-3 in 2005) and in Lahore (DENV-3 in 2008). A more recent study of Coo et al. 2013 [75] on genome sequencing of dengue serotypes has also documented that DENV-2 & 3 prevailing in Northern parts (Punjab & KPK) has common genetic ancestry with serotypes in Southern parts (Karachi). DENV serotype 3 (65%) dominated this outbreak followed by serotype 2 (35%) (Table 6). Previously we have observed DENV-2 (77%) and DENV-3 (22%) as responsible serotypes for the infection of *Aedes* mosquito in 2012 (Peshawar). In earlier studies, DENV-1, 2, 3 & 4 have been reported as main culprit during dengue epidemics in Pakistan [6, 76–78]. The current results thus have proved the hypothesis that the serotypes (DENV-2 & 3) detected in Swat may be the continuation of previous outbreaks and further the evidence of trans-ovarial transmission as a mean of dissemination of DENV in Pakistan. The prevailing/continuation of similar serotypes of dengue virus in the region since long show the significant role of trans-ovarial transmission of DENV in the *Aedes* mosquitoes. It is worth mentioning that we processed the mosquitoes irrespective of their sex and feeding. The presence of DENV in adult mosquitoes in our study shows; either the mosquito inherited the virus from its infected female parent or has taken the blood from the dengue infected patient.

The present findings predict that if the epidemics come next year with other than DENV-2 & 3 serotypes, the risk for DHF and DSS will increase in this region. Furthermore, our results also suggest that *Aedes* (reservoir of virus) if not eradicated effectively, may cause massive outbreaks in non-endemic areas of the region. The current study also suggest for the interventions to eliminate/control vector, breeding and immature by the implementation of three lines of action, e.g; environmental sanitation, education and training on community participation, use of environment friendly chemicals and biological agents. Entomological surveillance of dengue vectors for early action is the most important phase in the control of vector population.

These entomological and molecular investigations of the dengue outbreak (2013) revealed a high level of vector(s) infestation in the natural and man-made water holding containers in human dwellings as well as in public areas particularly during July, August and September, in parallel with the disease trend. In light of this research we concluded that there is an urgent need: (i) to educate people to adopt the improved water-storage practices (like proper covering of water-holding containers to prevent vectors breeding and personal protective measures, particularly during the rainy season to prevent the vectors- humans contact to reduce disease incidence); (ii) for the implementation of an integrated vector management practices; (iii) to constitute a separate “Dengue Control Cell” for strengthening mosquito nets on doors and windows, use of larvicides to eradicate the vector breeding sources, etc.; (iv) reduction measures of the vector population to eliminate unnecessary containers and properly seal the water reservoirs, as the dispersion of females outside the home is caused by the presence of preferred breeding sites; (v) for instructing the local administration on regular water supply and proper solid waste management; (vi) and also an advance research on vector mosquitoes and factors promoting the vector(s) growth/densities, disease epidemiology and characterization of DENV is the demand of today to reduce the spread of dengue in Pakistan.

## Supporting Information

S1 File The case investigation form which includes the whole information about the history of dengue patient, sign and symptoms of dengue, biochemical and serological information of dengue patient. (DOCX)
