# Supplementary material for: A Comprehensive Entomological, Serological and Molecular Study of 2013 Dengue Outbreak of Swat, Khyber Pakhtunkhwa, Pakistan
Source: PLoS One. 2016 Feb 5;11(2):e0147416. doi: 10.1371/journal.pone.0147416 (PMC4746065; doi:10.1371/journal.pone.0147416)
Supplement: S1 File — (DOCX) [file pone.0147416.s001.docx]

1. The data may kindly be available at access to the Zoology department, AWKUM, Bunir Campus, Khyber Pakhtunkhwa, Pakistan.
   Data can be fully accessed at [abu_amna2013@hotmail.com](mailto:abu_amna2013@hotmail.com)"
2. We do not have any ethical or legal restrictions to making our data publicly accessible.
